# Lateral Interbody Fusion for Treatment of Discogenic Low Back Pain: Minimally Invasive Surgical Techniques

**DOI:** 10.1155/2012/282068

**Published:** 2012-04-03

**Authors:** Luis Marchi, Leonardo Oliveira, Rodrigo Amaral, Carlos Castro, Thiago Coutinho, Etevaldo Coutinho, Luiz Pimenta

**Affiliations:** ^1^Instituto de Patologia da Coluna, São Paulo 04101-000, SP, Brazil; ^2^Department of Imaging Diagnosis, Universidade Federal de São Paulo, São Paulo 04024-002, SP, Brazil; ^3^Department of Neurosurgery, University of California, San Diego, CA 92103-8893, USA

## Abstract

Low back pain is one of the most common ailments in the general population, which tends to increase in severity along with aging. While few patients have severe enough symptoms or underlying pathology to warrant surgical intervention, in those select cases treatment choices remain controversial and reimbursement is a substancial barrier to surgery. The object of this study was to examine outcomes of discogenic back pain without radiculopathy following minimally-invasive lateral interbody fusion. Twenty-two patients were treated at either one or two levels (28 total) between L2 and 5. Discectomy and interbody fusion were performed using a minimallyinvasive retroperitoneal lateral transpsoas approach. Clinical and radiographic parameters were analyzed at standard pre- and postoperative intervals up to 24 months. Mean surgical duration was 72.1 minutes. Three patients underwent supplemental percutaneous pedicle screw instrumentation. Four (14.3%) stand-alone levels experienced cage subsidence. Pain (VAS) and disability (ODI) improved markedly postoperatively and were maintained through 24 months. Segmental lordosis increased significantly and fusion was achieved in 93% of levels. In this series, isolated axial low back pain arising from degenerative disc disease was treated with minimally-invasive lateral interbody fusion in significant radiographic and clinical improvements, which were maintained through 24 months.

## 1. Introduction (Succinct)

Intervertebral disc degeneration in the spine is natural process of aging and in many cases is asymptomatic [1]. However, low back pain (LBP) is strongly associated with lumbar disc degeneration [2]. LBP is one of the most common reasons for physician visits and loss of workplace productivity worldwide, thus the issue encompasses important clinic and socioeconomic consequences.

Conservative (nonoperative) care for LBP, while covering many different modalities, generally includes treatment with NSAIDs, weak opioids, and exercise therapy [3]. When extensive conservative therapies fail to adequately manage LBP, lumbar fusion is on possible surgical option, though its use remains controversial, as reported in the literature [4–8].

The objective of this work was to evaluate minimally invasive lateral interbody fusion in the surgical treatment of lumbar discogenic pain, and to perform a literature review of degenerative disc disease and its treatment in the literature.

## 2. Methods

Data were collected through retrospective review of prospectively collected clinical and radiographic registry at a single institution. Inclusion in the current study included consecutively treated patients with degenerative disc disease presenting with discogenic low back pain without radicular symptoms, after failing at least 6 months of conservative care. Discogenic pain was assessed by clinical examination [9], such as centralization phenomenon and pain during standing, and radiological signs of degeneration [10], such as black discs and endplate modifications. Provocative discography was not routinely used in making diagnostic conclusions. Patients with idiopathic/degenerative scoliosis or grade II/III/IV spondylolisthesis were excluded from the study. A psychological screening [11] was performed preoperatively, to assess psychosocial features, patient understanding and to adapt patient expectations according to the surgical objective.

Patients were treated via the minimally invasive, lateral retroperitoneal transpsoas approach [12]. The surgical procedure was performed with patients in a true 90° lateral decubitus position and the table was flexed to increase the distance between the iliac crest and the rib cage. Retroperitoneal blunt was used to dissect through the psoas muscle, using progressive dilators and an expandable retractor to expose the lateral surface of the spine. Real-time directional electromyography (EMG) with discrete-threshold responses was used in all cases (NeuroVision JJB System, NuVasive Inc, San Diego, CA). Wide discectomies were performed with release of the contralateral annulus while preserving the anterior and posterior longitudinal ligaments. Interbody spacers were placed on the lateral and posterolateral borders of the apophyseal ring to increase contact with strong cortical bone [13, 14], to restore disc height, sagittal and coronal plane alignment [15–18], and to indirectly decompress the neural structures [19]. The interbody grafts were made from polyetheretherketone and filled with recombinant human BMP-2 (Infuse, Medtronic Sofamor Danek, Memphis, TN), silicate substituted calcium phosphate (Actifuse ABX, Apatech, Hertfordshire, England), calcium sodium phosphate cement (Graftys HBS, Graftys, Aix-en-Provence, France), or hydroxyapatite (HAP-91, Implamed, Sao Paulo, Brazil).

Clinical evaluations were performed by a clinical and included a physical exam for lower extremity motor and sensory function and self-assessed questionnaires using the Oswestry disability index (ODI) and visual analogue scale (VAS) for back and leg pain. Evaluations were performed preoperatively and at 1 and 6 weeks, 3, 6, 12, and 24 months postoperative. Minimum follow-up for inclusion in the current analysis was 24 months postoperatively.

Bony fusion was assessed by two spine surgeons and two spine researchers in CT scans and dynamic X-rays. Fusion was considered complete when translational motion was <3 mm, angular motion was <5°, and >50% of disc space showed complete bony bridging.

Statistical analyses included descriptive statistics to characterize baseline variables and paired *t*-testing to evaluate differences in mean outcome variables from pre- to postoperative time points. Statistical analyses were performed using SPSS software (SPSS, Version 10, SPSS, Chicago, Ill, USA) and statistical significance was evaluated at *P* < 0.05.

## 3. Results

From 220 patients that underwent lateral interbody fusion for degenerative disc disease between August 2007 and December 2009, 22 (10%) patients met inclusion-exclusion criteria (mean age 57.6 years, range 32–85; mean BMI 28.9, SD 7.9; 50% female) with 28 spine levels treated. One- and two-level procedures were performed in 16 (73%) and 6 (27%) cases, respectively. Levels treated included L2-3, L3-4, and/or L4-5.

Surgical procedures were performed in an average of 72.1 minutes (range 40–110 min) with an average blood loss of less than 50cc. The average hospital discharge was 21 hours (range 8–44 hours). Intraoperative complications included one instance of anterior longitudinal ligament rupture, which resulted in the placement of posterior pedicle screws. No other intraoperative complications were observed. Three patients (5 spine levels) required supplemental percutaneous pedicle screw instrumentation for grade I spondylolisthesis with instability, while other cases (23 spine levels) were performed as stand-alone interbody constructs.

Four stand-alone levels experienced cage subsidence (14.3%) by 6-week followup. These patients experienced transient axial back pain (persisting several months) and in one (4.5%) case radiculopathy arose, which required a foraminotomy 12 months postoperative.

**Figure 1:**
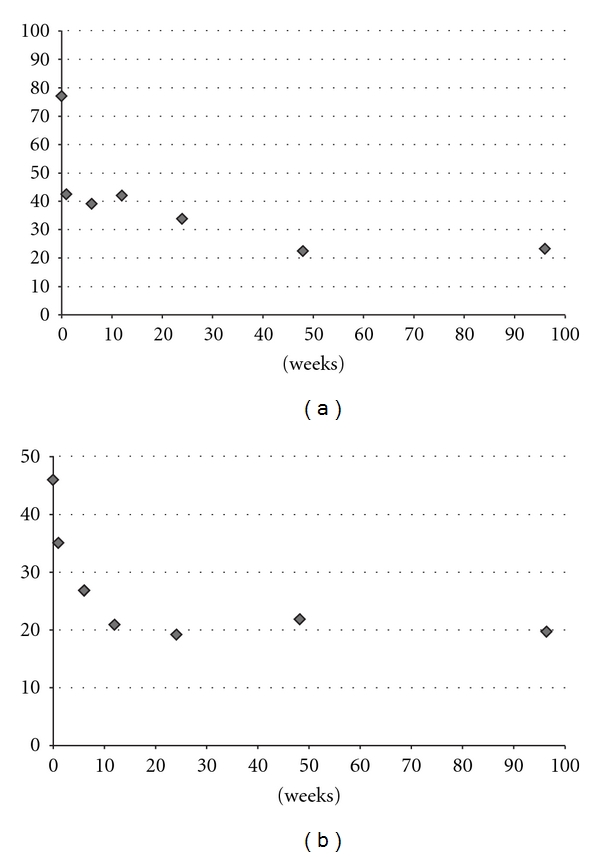
Clinical outcomes. (a) VAS back pain scores, all postoperative results are statistically significant compared to baseline (*P* < 0.003). (b) ODI scores, results are statistically significant since 1-week followup (*P* < 0.04) and in other postoperative visits (*P* < 0.001) compared to baseline.

Clinical outcomes improved postoperatively (Figure 1 and Table 1). LBP, assessed by VAS, showed a 44.2% improvement at the first postoperative visit (1 week) further improving to a 70.1% reduction at final followup. Disability was also significantly lowered as early as one week following surgery (24% improvement in ODI) and was further lowered until last followup, when a 52.5% improvement was observed (Figure 1). 

**Figure 2:**
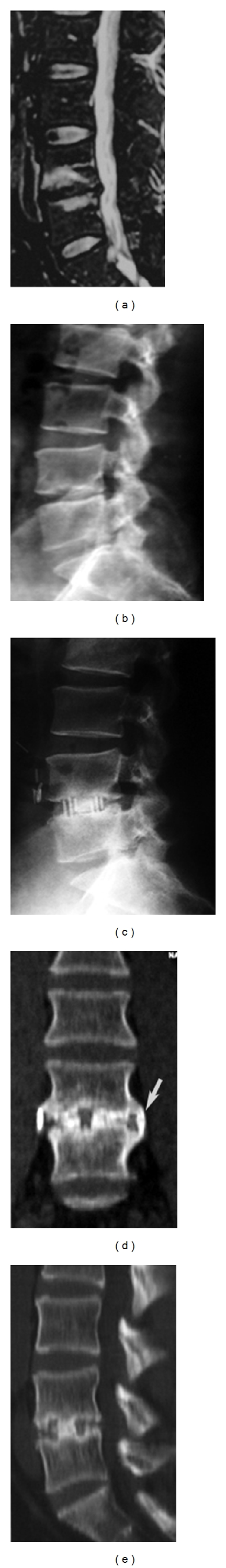
Case example number 1. Male, 54 years old, 7-year pain history which used to get worst by end of the day, refractory to physiotherapy and chiropractic. VAS scores-preoperative 8; 1-week 2; 24-month 1. Patient underwent an L4L5 stand-alone lateral interbody fusion. (a) Preoperative sagittal MRI. (b) Preoperative lateral orthostatic X-ray. (c) 24-month lateral orthostatic X-ray. (d) 24-month computed tomography coronal reconstruction, arrow shows fusion sentinel sign. (e) 24-month computed tomography sagittal reconstruction.

**Figure 3:**
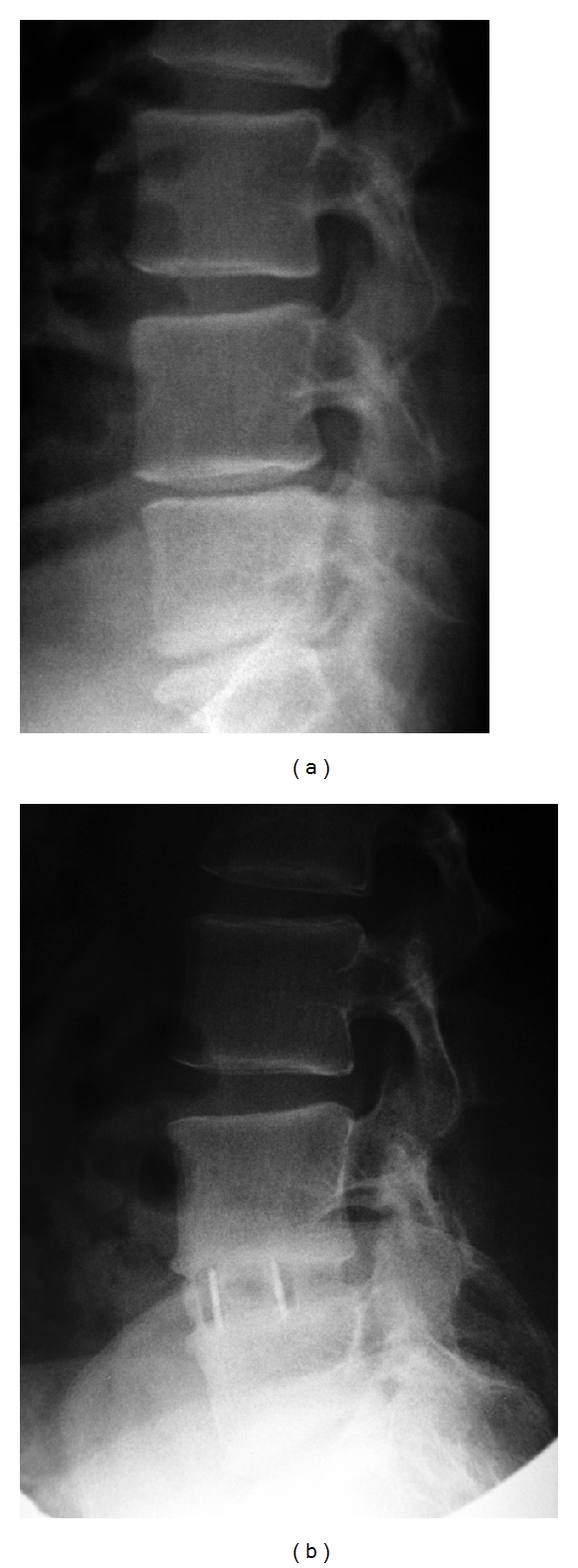
Case example number 2. Male, 58 years old, long history of lumbar axial pain and recurrent crisis event. VAS scores-preoperative 6; 1-week 3; 24-month 1. Patient underwent an L4L5 stand-alone lateral interbody fusion using rh-BMP. (a) Preoperative lateral orthostatic X-ray (b) 12-month lateral orthostatic X-ray.

Index level lordosis significantly changed from a mean preoperative value of 12.2° (7.4° SD) to 16.7° (6.5° SD) at final followup (*P* = 0.032). Bony fusion was observed in 92.9% (26/28) of total lumbar levels treated (exemplified in Figures 2 and 3). 

## 4. Discussion

This work examined the treatment of discogenic LBP in patients with degenerative disc disease treated with a discectomy and interbody fusion via lateral access. Isolated axial low back pain rapidly resolved after surgery and disability more gradually improved, as would be expected. Radiolographic analysis revealed improvements in segmental lordosis at treated levels and a high rate of solid fusion. Additionally, few complications occurred, as would be expected using a modern minimally invasive approach, and the patients were generally treated successfully through removal of the pathological intervertebral disc and by stabilizing and fusing the level.

This work represents a retrospective study on prospectively collected data in a small case series with midterm followup, so conclusions are limited to the study design drawbacks. The primary reason for a small sample size was the relative infrequency of surgical candidates for lumbar spine fusion surgery without radicular symptoms (only 10% of all cases in this series). This strengthens the results through sample homogeneity, but greatly limited the sample.

Intervertebral disc morphology continuously changes from birth to late stages of the human life [20]. Disc degeneration is a natural phenomenon, detectable in individuals as early as 11 to 16 years old. By the age of 50, approximately 10% of lumbar intervertebral discs would be classified as degenerated to some extent on MRI and severely degenerated in as many as 60% of 70-year-old discs [21, 22]. Macroscopical changes during this process have been described [23, 24]: the nucleus is the first to change and goes from exhibiting fluid-like to solid-like behavior; the annulus suffers a decrease in the number of layers, decrease in radial permeability, defects in the structure, and microfailure; subchondral bone/nucleus junction calcification, exhibition of focal defects and Modic changes culminate to display the ongoing inflammatory process.

Various phenomena are involved in lumbar disc disease. Genetics, trauma, nutrient pathways, cell death, and matrix synthesis can be primary degeneration inductors [24] and biomechanical matters also greatly contribute to the disease [25, 26]. Impaired neuromuscular control of the paraspinal and abdominal muscles (muscle hypo- or hyperfunctionality) and external forces (e.g., sustained and repetitive loading) can additionally cause disc damage [25, 26], to the point where only a narrow safe window remains between hypermobility (wear and tear) and underuse (immobilization).

Although in normal anatomy, intradiscal nerve terminations have a limited distribution (mostly on the posterolateral annulus), disc degeneration has been shown to have a massive ingrowth of nerves fibers [27–32]. These growths seem to penetrate from outside to inside the annulus, along the edges of annular fissures, dependent of the inflammation process and dependent upon specific markers like substance P and receptor to CGRP-ir nerve growth factor [29–31]. Nociceptive information is transmitted primarily by small neurons associated with inflammatory pain and some specific proinflammatory mediators (NGF; PGE2, IL-1, IL-6; IL-8) [29, 32, 33]. And importantly, these networks tend to resultantly function under peripheral and central sensitization [9, 29–31].

One of the most challenging factors of discogenic low back pain is an accurate differential diagnosis. Morphological and functional statuses of apophyseal joints, ligaments and musculature and spine biomechanics must be analyzed [9, 34–36]. Additionally, external forces and postural behavior also interfere in symptoms onset [25, 26]. Psychosocial factors such as depression, anxiety, and worker's compensation act an positive feedback in pain modulation and may be a drawback in diagnosis and treatment [11, 37–39].

Classically discs are innervated segmentally and discogenic pain pathways flow through the sinuvertebral nerve into the corresponding dorsal root ganglion and into the spinal cord, generating symptoms located at the index level [29, 40, 41]. More recently, an alternative pathway through the grey ramus communications has been described [41, 42]. The signal travels into the upper lumbar dorsal root ganglion (especially at the L2 level), when a L4-5 disc pathology may generate signals in an L2 dermatome, like a groin and anterior tight pain during a L4-5 provocative discography procedure [41, 42].

Identification of signs and symptoms of discogenic back pain includes continuous axial low back pain persistent in extended period deep in the central line of the spine, usually with no irradiation (few times with diffuse or inguinal irradiation), possible relief when lying, no significant worsening with movements, and worsened with axial load and long standing or sitting periods. In radiolographic analysis, low signal intensity of the disc on sagittal T2W, high-intensity zones, annular damages, and especially Modic changes corroborate clinical findings [9, 28, 36, 43].

Provocative discography is one of the possible tests to contribute in the diagnosis of discogenic pain, but a few studies have shown equivocal results for discography [44–46] and the procedure can also accelerate progression of degeneration changes in the lumbar disc [47]. False-positive rates were once reported to reach up to 40% [46], and the presence of many confounding factors can limit its potential: speed and pressure control; low/high pressure provocation; quiescent phase of the illness; somatization disorder; regular medications; abnormal psychometric scores; worker's compensation.

When a degenerated intervertebral disc is determined to be the primary pain generator, surgical removal must be considered. Nucleus replacement was one attempt to treat discogenic pain and maintain movement and function, but the ideal indication window is too narrow and several unwanted complications have occurred [48–52]. Lumbar fusion has been widely used for different pathological conditions resulting from idiopathic changes, degeneration, trauma, infection, or neoplasia. As reviewed elsewhere [53], lumbar fusion has more high-quality studies testifying favorable comparative outcomes [54–56] than with nonoperative care [57].

For a painful disc, discectomy and interbody fusion intend to remove the pathologic tissue, which presents itself as nonfunctional fibrotic structure, soaked with inflammatory mediators and nerve ingrowth, and to fuse the segment. Additionally, index motion is related to pain occurrence and can be treated with lumbar level stabilization, and the addition of interbody fusion has show the favorable results in lumbar fusion [56, 58, 59], especially for discogenic pain.

Lateral interbody fusion has been shown to significantly increase foramen and disc height [19], impact sagittal [60–62] and coronal plane reconstruction [15, 16, 18, 63, 64], and provide indirect decompression and relief of low back and irradiated symptoms [65, 66]. With true 90° lateral access, satisfactory results have also been shown in thoracic access for the treatment of tumor [67, 68], trauma [68], spondylolisthesis [61, 64], and disc herniation [69]. Moreover, artificial discs placed laterally have been an advance in lumbar arthroplasty due to anterior and posterior longitudinal ligament preservation [70].

If the affected lumbar level does not present with gross instability, a stand-alone interbody construction may be considered. In this instance, posterior muscle damage is prevented as well as posterior instrumentation complications. Biomechanical studies [71] have shown lateral interbody implants provide the largest reduction in range of motion in a stand-alone construct, with this stability increasing when moving from 18 mm cages (anteroposterior dimension), to wider ones (22 and 26 mm) [72].

Payment and reimbursement for lumbar fusion, especially for degenerative disc disease, are being rigorously reviewed by North American and worldwide institutions with the premise that it is ineffective. In this study, however, at 2 years postoperatively over 70% improvement in VAS and patient outcomes was demonstrated, much higher than previous studies on treatment for degenerative spine condition [55, 73–76]. This study, while somewhat limited, has shown that, in carefully selected patients, MIS lumbar fusion can be effective in treating isolated axial discogenic low back pain. The spine community must continue to debate the benefits and drawbacks of lumbar fusion for degenerative disc disease.

## Figures and Tables

**Table 1:** Clinical and radiological results.

	Preop	6 weeks	*P* value	24 months	*P* value
VAS (cm)	7.7 ± 2.4	4.3 ± 2.2	0.001*	2.3 ± 1.9	<0.001*
ODI (%)	46 ± 19	27 ± 14	<0.001*	19.6 ± 13	0.003*
Segmental Lordosis (degrees)	12.2° ± 7.4°	—	—	16.7° ± 6.5°	0.031*
Fusion	—	—	—	92.9% (26/28)	—

*P* Values are referent to comparison to Preop values. *Statistically significant.
